# Wild Boars as Hosts of Human-Pathogenic *Anaplasma phagocytophilum* Variants

**DOI:** 10.3201/eid1812.120778

**Published:** 2012-12

**Authors:** José de la Fuente, Christian Gortazar

**Affiliations:** Author affiliations: Instituto de Investigación en Recursos Cinegéticos, Ciudad Real, Spain (J. de la Fuente, C. Gortazar);; Oklahoma State University, Stillwater, Oklahoma, USA (J. de la Fuente)

**Keywords:** pig, boar, rickettsia, zoonosis, reservoir, epidemiology, bacteria, Anaplasma phagocytophilum, transcriptomics

**To the Editor:** Michalik et al. (*1*) reported a 12% prevalence of *Anaplasma phagocytophilum*, the causative agent of human granulocytic anaplasmosis and tick-borne fever of ruminants, in wild boars in Poland. *A. phagocytophilum* has been reported with low prevalence among wild boar in the Czech Republic, Slovenia (*2*), and Japan (*3*). In Spain and Mississippi, United States, *A. phagocytophilum* in wild boars or feral pigs, respectively, has not been reported (*4*,*5*). Furthermore, in Slovenia and Poland, the *A. phagocytophilum* gene sequences found in samples from wild boars were identical to those found in samples from humans and the tick vector *Ixodes ricinus* (*1*). These results suggested, as pointed out by Michalik et al. (*1*), that wild boar might play a role in the epizootiology of *A. phagocytophilum* by serving as a natural reservoir host, at least in some regions.

To test this hypothesis, we conducted transcriptomics studies to characterize host response to *A. phagocytophilum* infection in naturally and experimentally infected boars (*6*,*7*). The results suggested that boars are susceptible to *A. phagocytophilum*, but are able to control infection, mainly through activation of innate immune responses and cytoskeleton rearrangement to promote phagocytosis and autophagy. Control of *A. phagocytophilum* infection in boars might result in infection levels below PCR detection or infection clearance, contributing to the low percentage of infection prevalence detected for this species in most regions.

The low detection levels suggest that boars have a low or no impact as a reservoir host for *A. phagocytophilum*. Even if boars remain persistently infected with *A. phagocytophilum* at low levels by downregulating some adaptive immune genes and delaying the apoptotic death of neutrophils through activation of the Jak-STAT pathway, among other mechanisms (*6*), their role as a source of infection for ticks remains to be demonstrated.
